# The emerging role of olanzapine in paediatric CINV control: A review

**DOI:** 10.1097/MD.0000000000032116

**Published:** 2022-12-16

**Authors:** Anabella Karla Barušić

**Affiliations:** 1 The datasets generated during and/or analyzed during the current study are publicly available. a The Psychoncology Team, Department of Child & Adolescent Psychological Medicine, University Collage Hospital, London, UK.

**Keywords:** chemotherapy-induced nausea and vomiting (CINV), paediatric oncology, olanzapine, antiemetics, chemotherapy

## Abstract

Chemotherapy-induced nausea and vomiting (CINV) is a serious side effect of chemotherapy that negatively impacts the quality of life of oncological patients and is associated with the emetogenic risk specific to administered chemotherapy. Current practice guidelines on the use of antiemetics in CINV include the option of adding olanzapine to antiemetic regimens in the management of adult CINV. The use of olanzapine in pediatric CINV has been restricted to children with poor CINV control. Research on the use of olanzapine in pediatric CINV has been limited. The aim of this review was to evaluate current evidence on the effective and safe antiemetic use of olanzapine in pediatric CINV of any type following chemotherapy of any emetogenicity. Ovid MEDLINE, Embase, CENTRAL databases were searched for any literature on the use of olanzapine in pediatric CINV published from 2015 to 2022. Studies that reported on the olanzapine-containing antiemetic regimen in peadiatric CINV control specifically were included. Search restrictions were placed on research published in English. The search generated 43 records that were assessed for eligibility. Out of 10 identified eligible studies a third were RCT. Findings of this review suggest that adding olanzapine to antiemetic regimen in pediatric CINV control is a worthwhile consideration. Further research is needed to establish the efficacy and safety of antiemetic olanzapine use in pediatric CINV.

## 1. Introduction

Chemotherapy-induced nausea and vomiting (CINV) is a serious side effect of chemotherapy that negatively impacts the quality of life of oncological patients.^[1,2]^ The risk of CINV is associated with the emetogenic risk (high, moderate, low and minimal) specific to administered chemotherapy.^[3]^ The classification of CINV is summarized in Table 1.

**Table 1:** Classification of CINV.^[17–21]^

**Type of CINV**	**Description**
Acute	CINV that occurs within 24 h of chemotherapy administration
Delayed	CINV that occurs after 24 h of chemotherapy administration; often peaks between 24 and 72 h
Breakthrough	CINV that occurs within 5 d of chemotherapy administration despite optimal antiemetic regimen and requires rescue medication
Refractory	CINV that occurs in subsequent chemotherapy cycles despite maximum antiemetic protocol
Anticipatory	CINV is triggered sensory stimuli (smell, sound, taste) associated with chemotherapy administration; previous experience of CINV

CINV =chemotherapy induced nausea and vomiting.

Nausea and vomiting are part of protective reflexes that clear the gastrointestinal system of toxins. The act of vomiting occurs when afferent nerve fiber impulses travel from the gastrointestinal tract, cerebral cortex and chemoreceptor trigger zone (CTZ) in the medulla oblongata to the vomiting center (VC) located within the same brainstem structure. This in turn triggers efferent nerve fiber impulses to travel back to different parts of the gastrointestinal and respiratory system, resulting in vomiting. Chemotherapy agents are thought to activate neurotransmitter receptors in the gastrointestinal tract, CTZ and VC and affect areas in the cerebral cortex, medulla oblongata and the small intestine via the vagus nerve.^[3]^ Gastrointestinal and central nervous system neurotransmitters that mediate the afferent inputs to VC include serotonin, dopamine, substance *P* and acetylcholine.^[4]^ The introduction of agents that act as neuroreceptor antagonists in CTZ, VC and the gastrointestinal tract have shown to be effective in pharmacological management of CINV (Table 2).

**Table 2:** Antiemetic agents used in pediatric CINV according to receptor antagonism.^[1–3,22–25]^

Dopamine receptor antagonists	5-HT3 receptor antagonists	Dopamine - 5-HT3 receptor antagonist properties	NK1 receptor antagonists
Droperidol	Azasetron	Metoclopramide***	Aprepitant
Olanzapine	Dolasetron*		Fosaprepitant
Haloperidol	Granisetron		Netupitant
Prochlorperazine**	Olanzapine		Rolapitant
	Ondansetron*		
	Palonosetron		
	Ramosetron		
	Tropisetron		

CINV = chemotherapy-induced nausea and vomiting, NK1 = neurokinin, *5-HT3 =* serotonin.

* intravenous administration associated with increased risk of QT interval prolongation ^[26,27]^.

** no longer recommended for CINV prophylaxis due to persistent and potentially life-threatening adverse events ^[23,25]^.

*** dose dependent antagonism i.e. dopaminergic at lower doses and serotonergic at higher doses ^[28]^.

Olanzapine is a second-generation atypical antipsychotic of the thienobenzodiazepine class routinely used in psychiatric practice for the treatment of psychotic disorders, but has many off-label uses. Olanzapine has a unique receptor profile amongst antipsychotics, which in part accounts for its various uses.^[5]^ Due to its antidopaminergic, antiserotonergic and anticholinergic properties, olanzapine has shown efficacy for the treatment of emesis, delirium, anxiety, insomnia, anorexia and cachexia in adults.^[6–8]^ Olanzapine blocks dopamine and serotonin receptors that are recognized CINV mediators and has recently been included in international guidelines on the use of antiemetics in management of CINV.^[9,10]^ Olanzapine has a relatively favorable safety profile for extrapyramidal side effects and QTc prolongation and less favorable for drowsiness^[11]^ and weight gain ^[12,13]^ when compared to other antipsychotics. The weight gain is more apparent in children taking olanzapine compared to adults.^[14]^ The dosing schedules and safety profile of olanzapine has not been well-studied in children below 13 years of age.^[15]^

Notwithstanding the growing efficacy of newer antiemetic agents, CINV remains a significant complication of chemotherapy.

Current clinical practice guidelines (CPG) published in 2016 by the American society of clinical oncology and multinational association of supportive care in cancer/European Society of Medical Oncology on the use of antiemetics in CINV include the option of adding olanzapine together with a 5-HT3 receptor agonist, dexamethasone and a NK1 receptor antagonist in adults receiving high emetogenic chemotherapy (HEC). Furthermore, the 2016 CPG make a conditional recommendation for the use of olanzapine in pediatric patients receiving HEC who experience breakthrough CINV. A conditional recommendation is also made for the use of olanzapine in pediatric patients receiving HEC with refractory CINV who cannot receive other antiemetics. This CPG identifies the optimal dose, efficacy, and safety of olanzapine as an evidence gap.^[16]^ Therefore, this review aims to evaluate current evidence on the antiemetic use of olanzapine in pediatric patients receiving chemotherapy of any emetogenicity. The review focused on the efficacy and safety of olanzapine in the control of pediatric CINV of any type.

### 1.1. Study selection

This review included research articles published from January 1, 2015 to October 1, 2022. We made a pragmatic decision to only include studies published from 2015 onwards since this is the year when the first study evaluating the addition of olanzapine to standard antiemetic regimen in control of pediatric CINV was published. Initially we set out to include double-blinded randomized control trials (RCT) only however due to paucity of generated results we decided to include open label RCT, feasibilities studies, prospective and retrospective observational studies. Studies focusing on pediatric patients (≤18 years of age) receiving chemotherapy of any emetogenicity (HEC, Moderate emetogenic chemotherapy [MEC], low emetogenic chemotherapy) with any type of CINV (acute, delayed, anticipatory, breakthrough, refractory) were included in the review. Only studies that reported on olanzapine-containing antiemetic regimens to control pediatric CINV were included. Studies with adult oncological patients (>18 years of age) were excluded. Abstracts and letters to the editor were excluded.

## 2. Methodology

A literature search was conducted to identify any studies on the use of olanzapine in the control of pediatric CINV. For the purpose of this review Ovid MEDLINE, Embase, CENTRAL were searched using MeSH terms “pediatric oncology,” “children,” “adolescents,” ’chemotherapy-induced nausea and vomiting’ and “olanzapine” on 1 March, 9 May and 1 October 2022 respectively. Search restrictions were placed on studies published in the English language.

## 3. Results

**Figure 1.**
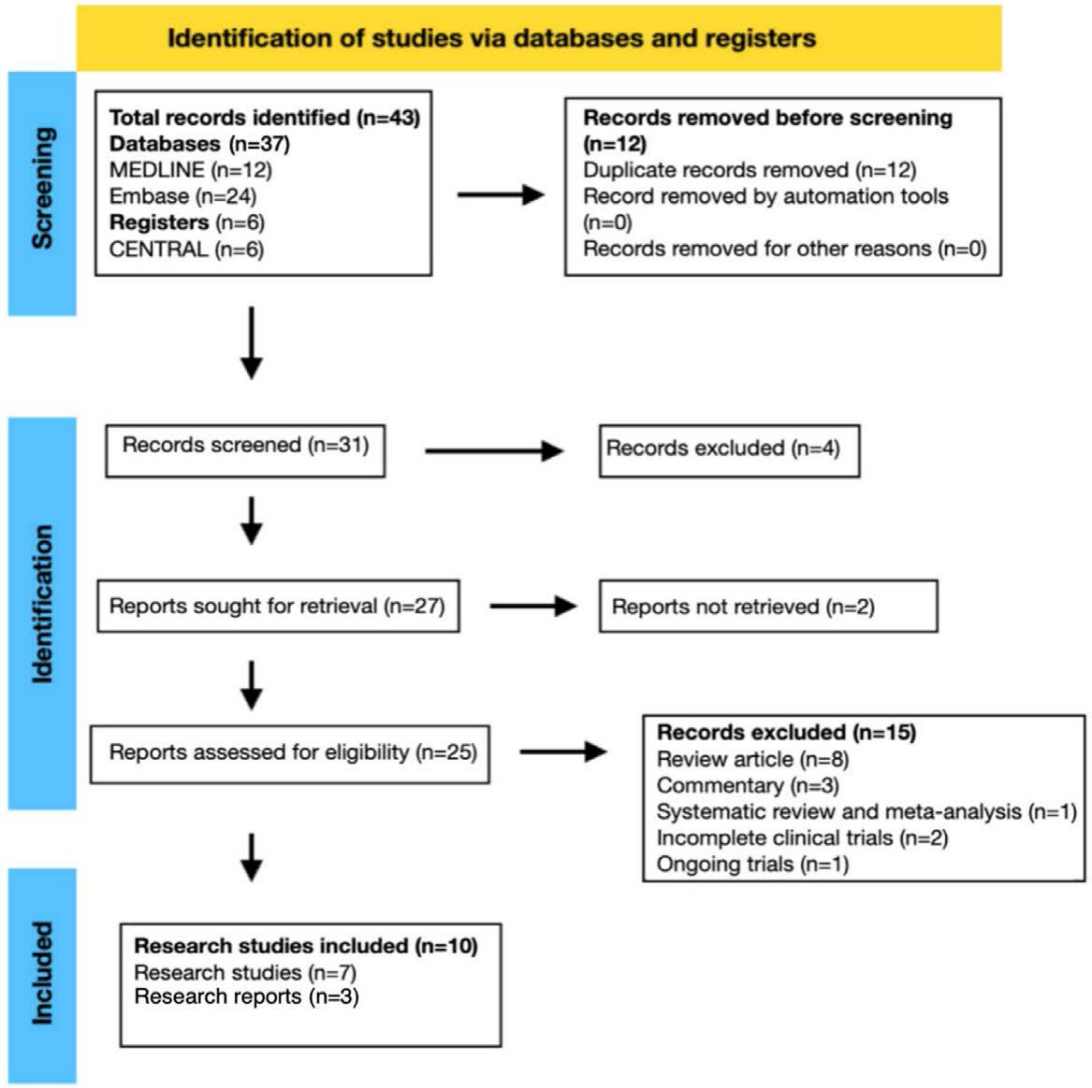
Study flow diagram.

The search generated 43 records and after removal of duplicates 31 records underwent level 1 screening (study title and abstract). Only studies that reported on the olanzapine-containing antiemetic regimen for management of pediatric CINV were included. Records that reported on adult CINV management (2 records), non-olanzapine containing regimen (2 records) and had fewer than 10 participants were excluded. Level 2 (full text) screening was possible for 25 reports as 2 reports could not be retrieved in time after contacting the study authors. Reference lists of included articles were also assessed to identify other potentially relevant studies. Reports that evaluated the efficacy and safety of olanzapine-containing antiemetic regimen for prophylaxis and treatment of pediatric CINV and chemotherapy-induced vomiting (CIV) were assessed for eligibility. Due to the limited availability of evidence, comparator, non-comparator, pilot/feasibility and observational longitudinal studies were assessed for eligibility. Editorials, commentary, research letters, narrative reviews, systematic reviews, meta-analysis and incomplete clinical trial studies were excluded (15 reports). Eligible research articles and research reports were included in this systematic review (Fig. 1).

### 3.1. Study characteristics

Individual characteristics of identified studies are summarized in Table 3.

**Table 3:** Characteristics of included studies.

**Study/Year/Country**	**Sample** **N = no of participants (Age range**)	**Emetogenicty**	**Antiemetic regimen**	**Comparator**	**CINV control**
Moshayedi et al^[31]^ (2021) [Iran]	N = 40 (4–14yr)	MEC	OLZ + 5HT3RA + dexamethasone	Placebo + 5HT3RA + dexamethasone	Acute Delayed Overall
Naik et al^[32]^ (2020) [India]	N = 240 (5–17yr)	HEC	OLZ + aprepitant + 5HT3RA + dexamethasone	Placebo + aprepitant + 5HT3RA + dexamethasone	Acute Delayed Overall
Radhakrishnan et al^[33]^ (2020) [India]	N = 80 (5–18yr)	HEC MEC	OLZ	Metoclopramide	Breakthrough
Long et al^[34]^ (2015) [United States]	N = 14 (5–18yr)	HEC	OLZ + 5HT3RA + dexamethasone	Aprepitant + 5HT3RA + dexamethasone	Acute Delayed Overall
Flank et al^[35]^ (2018) [Canada]	N = 15 (4 - 17yr)	HEC MEC	OLZ + 5HT3RA ± dexamethasone ± aprepitant		Acute Delayed Overall
Samaddar et al^[36]^ (2017) [India]	N = 10 (median age 13yr)	HEC MEC	OLZ + 5HT3RA + dexamethasone		Acute
Teotia et al^[37]^ (2020) [India]	N = 31 (2 - 18yr)	HEC MEC	OLZ antiemetic regimen		Breakthrough
Lee et al^[38]^ (2020) [South Korea]	N = 12 (mean age 14.1 ± 5.5yr)	HEC MEC	OLZ antiemetic regimen		Acute Delayed Overall
Flank et al^[39]^ (2015) [Canada]	N = 60 (3–17yr)	HEC MEC LEC	OLZ antiemetic regimen		Acute
Davis et al^[40]^ (2018) [United States]	N = 121 (≤18yo)	HEC	OLZ antiemetic regimen		

RCT = randomized controlled trial, CR = complete response, HEC = high emetogenic chemotherapy, MEC = moderate emetogenic chemotherapy, LEC = low emetogenic chemotherapy, OLZ = olanzapine, 5HT3RA = 5HT3 receptor agonist, CINV = chemotherapy induced nausea and vomiting, CIN = chemotherapy induced nausea, CIV = chemotherapy induced vomiting.

### 3.2. Main results of individual studies

The main results of individual studies are summarized in Table 4.

**Table 4:** Summary of main results of individual studies.

**Study**	**Type of CINV**	**Emetogenicty**	**Comparison**	**CR (CIV**)	**CR (CIN**)	**Olanzapine toxicity**
Moshayedi et al^[31]^	Acute Delayed Overall**	MEC	OLZ vs placebo in addition to 5HT3RA + dexamethasone	MAT/CTCAE: 86.5%/81.25% vs 84%/94.7% *P* > .05*	MAT/CTCAE: 62.5%/68.7% vs 89.5%/79% *P* > .05**	Drowsiness: OLZ > placebo Constipation: OLZ > placebo
Naik et al^[32]^	Acute Delayed Overall	HEC	OLZ vs placebo in addition to aprepitant + 5HT3RA + dexamethasone	Acute: 78% vs 59% *P* = .001 Delayed: 74% vs 47% *P* < .001) Overall: 64% vs 38% *P* < .001	Acute: 74% vs 52% *P* < .001 Delayed: 74% vs 47% *P* < .001 Overall: 64% vs 37% *P* < .001	Drowsiness: OLZ vs placebo 35% vs 11% *P* < .001
Radhakrishnan et al^[33]^	Break-through	MEC HEC	OLZ vs Metoclopramide antiemetic regimen	72% vs 39% *P* = .003	59% vs 34% *P* = .026	Hyperglycaemia: OLZ > Metoclopramide Drowsiness: OLZ > Metoclopramide
Long et al^[34]^*	Acute Overall	HEC	OLZ vs aprepitant in addition to 5HT3RA + dexamethasone	Acute: 78.6% vs 76.9% Overall: 28.6% vs 23,1%	Overall: 1.87/15.3 vs 1.95/21.5	Agitation: OLZ only 13.3%
Flank et al^[35]^*	Acute Delayed	MEC HEC	OLZ + 5HT3RA ± dexamethasone ± aprepitant	Acute: 86 ± 21% Delayed: 75 ± 32%	Acute: 40 ± 39% Delayed: 50 ± 33%	Drowsiness (dose-dependent)
Samaddar et al^[36]^*	Acute	MEC HEC	OLZ + 5HT3RA + dexamethasone	HEC: 51.6% MEC: 66.67%		Drowsiness: 64.86% Constipation: 13.5%
Teotia et al^[37]^	Break-through	MEC HEC	OLZ antiemetic regime	80.9%		Drowsiness Elevated ALT/AST
Lee et al^[38]^	Acute Delayed Overall	MEC HEC	OLZ antiemetic regimen	75.6%		Drowsiness Hyperglycaemia Fatigue Disturbed sleep
Flank et al^[39]^	Acute	LEC MEC HEC	OLZ antiemetic regimen	65%		Drowsiness (dose-dependent)

CINV *=* chemotherapy induced nausea and vomiting, CIV = chemotherapy induced vomiting, CR = complete response, HEC = high emetogenic chemotherapy, MEC = moderate emetogenic chemotherapy, LEC = low emetogenic chemotherapy, OLZ = olanzapine, 5HT3RA = 5HT3 receptor agonist, MAT = multinational association for supportive care in cancer (MASCC) Anti-emesis Tool, CTCAE = common toxicity criteria for adverse events.

* Feasibility/pilot study.

** There was no statistical difference in outcome measures between study groups.

*NB:* Davis et al results are described elsewhere due to the nature of the study (evaluation of prescribing trends over time).

#### 3.2.1. Comparator trial studies.

In the last 7 years (from 2015–2021) there were 3 RCT assessed the efficacy and safety of olanzapine in prevention and treatment of pediatric CINV^[29–31]^ that all originated from low and middle income countries.^[12]^ These superiority trials comparing the effectiveness and safety of olanzapine to another drug in addition to standard antiemetic regimen in the prevention or treatment of pediatric CINV.

##### 3.2.1.1. Olanzapine versus placebo/no treatment.

Moshayedi et al double blinded superiority trial compared the efficacy and safety of adding olanzapine or a placebo to ondansetron and dexamethasone antiemetic regimen in children receiving MEC. Olanzapine was given at 0.14 mg/kg/dose (maximally 10 mg per day). The primary objective was to compare complete response (CR) rates (no vomiting and nausea and no rescue medication) in the acute (0–24 hours post-chemotherapy) or delayed phase (24–72 hours post-chemotherapy). CR was measured using the multinational association of supportive care in cancer Antiemesis Tool (MAT) and the common toxicity criteria for adverse events (CTCAE) grading system.^[39,40]^ The safety profile of olanzapine was the secondary objective of the study. A higher portion of CR was observed in the placebo group compared to olanzapine group for vomiting [84% vs 86.5% (MAT) and 94.7% vs 81.25% (CTCAE), *P* > .05] and for nausea [89.5% vs 62.5% (MAT) and 79% vs 68.7% (CTCAE), *P* > .05]. There was no statistical difference between acute or delayed CINV between groups. The frequency of adverse effects was higher in the olanzapine group (drowsiness and constipation). Moshayedi et al study did not prove superiority of olanzapine compared to placebo in the prevention or treatment of pediatric CINV. The significance of trial findings is arguable due to their lack of statistical power and external validity.

Similarly, an open label single-center RCT by Naik et al compared the efficacy and safety of olanzapine versus placebo in addition to aprepitant, ondansetron and dexamethasone in management of pediatric CINV. The experimental group received oral olanzapine 0.14 mg/kg/day (rounded to the nearest 2.5 mg; maximum, 10 mg) during the chemotherapy block and 3 days post-chemotherapy. The primary objective was to compare CR rates (no vomiting and no rescue medication) in the acute (0–24 hours post-chemotherapy), delayed (24–120 hours post-chemotherapy), and overall periods (0–120 hours post-chemotherapy). Nausea comparison was the secondary objective assessed by the edmonton symptom assessment scale.^[41]^ Edmonton symptom assessment scale is generally used in the adult population; thus, the use of a pediatric-specific nausea assessment tool would have been more appropriate. Safety comparison was an additional objective. CR for vomiting was significantly higher in olanzapine compared with placebo group in the acute (78% vs 59%; *P* = .001), delayed (74% vs 47%; *P* < .001) and overall period (64% vs 38%; *P* < .001). Similarly, CR for nausea was significantly higher in olanzapine compared with placebo group in the acute (74% vs 52%; *P* < .001), delayed (74% vs 47%; *P* < .001), and overall period (64% vs 37%; *P* < .001). Drowsiness was more commonly seen in the olanzapine group (35% vs 11%; *P* < .001). The trial demonstrated superiority of adding olanzapine to standard antiemetic treatment in management of pediatric CINV, compared to placebo. However, the open label and single center study design increases the risk of investigator led bias and compromises external validity.

##### 3.2.1.2. Olanzapine vs metoclopramide.

A single-center open label phase III trial by Radhakrishnan et al compared the efficacy and safety of olanzapine versus metoclopramide in the treatment of breakthrough CINV in children receiving MEC/HEC. Olanzapine was given orally as a tablet form (2.5 mg and 5 mg) in a weight-based dosing schedule (10–20 kg; olanzapine 2.5 mg/every 24 hours and > 20 kg; olanzapine 5 mg/every 24 hours). Metoclopramide was given orally (oral solution: 1 mg/mL and 10 mg tablet) in a weight-based schedule (10–35 kg; metoclopramide 0.15 mg/kg/dose q 8 hourly and > 35kg and > 14 years; metoclopramide 10 mg q 8 hourly). The primary objective was to compare CR rates (no vomiting and no rescue medication) between the groups in the delayed phase (72 hours post-chemotherapy). Nausea and drug toxicity were secondary study objectives. CR rates were significantly higher in the olanzapine group compared with the metoclopramide group for vomiting (72% vs 39%, *P *= .003) and nausea (59% vs 34%, *P *= .026). Hyperglycaemia and drowsiness were more commonly seen in the olanzapine group. The trial findings on superiority of olanzapine, compared to metoclopramide in treating pediatric breakthrough CINV failed to reach statistical significance. A single center open label study design increases the risk of observer bias and compromises external validity.

##### 3.2.1.3. Olanzapine vs aprepitant.

An open-label randomized cross-over feasibility trial by Long et al compared the efficacy and safety of olanzapine vs aprepitant in addition to ondansetron and dexamethasone in management of CINV in children receiving HEC. Participants received either aprepitant or olanzapine in first cycle of chemotherapy and then crossed over in second cycle of chemotherapy. Dosing for olanzapine (> 60 kg; olanzapine 10 mg orally daily for 4 doses, 40–59.9 kg; olanzapine 5 mg orally daily for 4 doses, 20–39.9 kg; olanzapine 2.5 mg orally daily for 4 doses, <20 kg; olanzapine 1.25 mg orally daily for 4 doses) and for aprepitant (> 40 kg; aprepitant 125 mg orally on day 1, then 80 mg orally daily on days 2 and 3, 35–39.9 kg; aprepitant 80 mg orally daily for 3 doses, 20–34.9 kg; aprepitant 40 mg orally daily for 3 doses, < 20 kg; aprepitant 1.5–2 mg/kg orally daily for 3 doses). The primary objective of this study was to determine the feasibility of recruitment and data collection for conducting a larger trial. Additional outcomes were the comparison of CR rates (no vomiting and no use of rescue medication), the absence of nausea and number of adverse drug events between the groups in the acute (0–24 hours post-chemotherapy) and overall (0–120 hours post-chemotherapy) phase. Nausea was measured by both patients and their caregivers using the baxter animated retching faces (BARF) pictorial scale (0–10) and the visual analogue scale (VAS) (0–100), respectively.^[42,43]^ Good control of nausea was set to be a score < 25 on VAS by parents and < 2 on BARF by patients. CR rates were higher in the olanzapine trial arm for vomiting in acute (78.6% vs 76.9%) and overall (28.6% vs 23,1%) phase. Patient and parent nausea ratings were lower in olanzapine compared to aprepitant group (1.87 vs 1.95 mean BARF score and 15.3 vs 21.5 mean VAS score). Agitation was seen in the olanzapine group only (13.3%). The trial demonstrated superiority of olanzapine, compared to aprepitant in the management of pediatric CINV. A larger RCT is needed to confirm the study findings.

#### 3.2.2. Non-comparator studies.

A prospective open-label, single arm, multicentre, feasibility study by Flank et al ^[33]^ assessed the efficacy and safety of olanzapine in addition to either ondansetron, granisetron, palonosetron ± dexamethasone ± aprepitant for management of CINV in children receiving MEC and HEC. All patients received oral olanzapine (0.12 ± 0.03 mg/kg/dose; max 10 mg/dose) once daily starting before the first chemotherapy dose and continuing for up to 4 doses after the last chemotherapy administration. The primary endpoint was the feasibility of patient recruitment and data collection for conducting a larger pediatric trial in the future. CIV, chemotherapy-induced nausea (CIN), and overall CINV control in acute (0–24 hours post-chemotherapy), delayed (24–201 hours post-chemotherapy) and overall phase (0–201 hours post-chemotherapy) were described as secondary endpoints. CIN was measured using the paediatric nausea assessment tool.^[44]^ Olanzapine toxicity was an additional outcome measure. CR (CIV) rate was 86 ± 21% and CR (CIN) was 40 ± 39% in acute phase. CR (CIV) was 75 ± 32% and CR (CIN) was 50 ± 33% in delayed phase. All study participants achieved CR (CIV) overall however CR (CIN) was achieved in only 1 study participant. Sedation occurred in 6 participants but resolved with olanzapine dose reduction. The trial demonstrated effectiveness of olanzapine-containing antiemetic regimen in prophylaxis of pediatric CINV. However, lack of a comparator group makes it impossible to discern the contribution of olanzapine in CINV control. The strengths of this study were its multi-center design, careful titration of the olanzapine dose against sedation, and the use of a validated pediatric nausea severity assessment tool. A larger RCT is needed to confirm the study findings.

#### 3.2.3. Observational studies.

A single center prospective observational study by Samaddar et al ^[34]^ evaluated the efficacy and safety of olanzapine in addition to ondansetron and dexamethasone in prevention of acute CINV in children receiving MEC and HEC. Olanzapine (0.1 mg/kg q 24-hourly) was prescribed from day 1 of chemotherapy. Nausea was assessed using a questionnaire. CR was classified as complete control of CINV (no nausea and vomiting) in acute phase. Olanzapine toxicity has also been assessed. CR (CINV) was achieved in 51.6% of patients receiving HEC and in 66.67% in patients receiving MEC. Sedation (64.86%) and constipation (13.5%) were the most common drug effects reported.

A similar study was conducted by Teotia et al ^[35]^ evaluating the efficacy and safety of olanzapine in treatment of breakthrough CINV in children receiving MEC and HEC. CR was classified as complete control of CINV (no nausea and vomiting) in acute phase. Olanzapine toxicity has also been assessed. CR were observed in 80.9% chemotherapy blocks, while 2.4% patients experienced refractory vomiting. The mean dose of olanzapine in patients with CR was 0.09 ± 0.02 mg/kg/dose. There was no statistical difference in CR rates based on age (<10/>10 years, *P* = .23), gender (*P* = .68), emetogenic regimen (MEC/HEC, *P* = 1.0) or single/multiple-day chemotherapy (*P* = .2). The most commonly reported adverse events were sedation in 9 patients and increased serum transaminase levels in 3 patients.

An observational retrospective single-center study by Lee et al ^[36]^ evaluated the safety and efficacy of olanzapine in the management of CINV in children receiving MEC/HEC. The primary endpoint was CR (no vomiting and no nausea and no use of rescue medication). Drug toxicity was a secondary endpoint. The mean dose was 0.07 ± 0.04 mg/kg/dose and 2.50 ± 1.37 mg/kg/dose for MEC and HEC, respectively. CR (no nausea, no vomiting and no use of rescue medication) was achieved in 75.6% of patients. Adverse effects included somnolence, hyperglycemia, fatigue, and disturbed sleep.

A retrospective observational multi-center study by Flank et al ^[37]^ evaluated the efficacy and safety of olanzapine in the prevention of acute CINV in children receiving low emetogenic chemotherapy/MEC/HEC. CR was classified as complete control CIV (no vomiting and no use of rescue medication). Toxicity was graded using CTCAE. Olanzapine was most often (59%) initiated due to a history of poorly controlled CIV. The mean initial single olanzapine dose administered was 0.10 ± 0.051 mg/kg/dose. The maximum single dose administered was 10 mg. Most children who received olanzapine beginning on the first day of the chemotherapy block experienced complete CIV control throughout the acute phase (65%). The study found no strong association between the dose/kg of olanzapine and the level of CIV control (OR 1.01; 95% CI: 0.999–1.020; *P* = .091). Sedation was reported in 7% of chemotherapy blocks and was significantly associated with increasing olanzapine dose (OR: 1.17; 95% CI: 1.08–1.27; *P* = .0001).

A retrospective observational single center report by Davis et al ^[38]^ examined the frequency of olanzapine prescribing as an antiemetic regimen for CINV management in children receiving HEC. For chemotherapy encounters from 2015 to 2017 (n = 262), the most commonly prescribed regimens were a combination of ondansetron and olanzapine (22%), ondansetron containing antiemetic regimen (22%), ondansetron only (18%), and a combination of ondansetron and aprepitant (13%). This was in contrast to chemotherapy encounters from 2013 to 2014 (n = 75) where the most commonly prescribed regimens were a combination of ondansetron and aprepitant (36%), ondansetron (oral and intravenous form 23%, intravenous form 17%) and a combination of ondansetron, aprepitant and dexamethasone (11%). The frequencies of specific antiemetic regimens differed significantly in 2015 to 2017 versus 2013 to 2014 (*P < *.001).

## 4. Discussion

The findings of this review help demonstrate the paucity of high-quality evidence on the use of olanzapine in pediatric CINV control. Research challenges present in pediatric CINV studies might help explain such findings and are discussed below.

First, the assessment of nausea and vomiting in pediatric CINV is not straightforward. This review identified high level of variability of assessment tools used to measure specific endpoints in pediatric CINV studies. The assessment and measurement of nausea is often problematic due to its subjective nature. Additionally, nausea can be part of a prodromal phase of vomiting ^[4]^ but it can also occur without vomiting.^[3]^ Thus, it is not surprising that a number of CINV studies use terms “no significant nausea” or “only mild nausea” to describe their outcome measures.^[26]^ CINV studies use psychometric scales to measure the severity of nausea. paediatric nausea assessment tool and BARF are validated measuring tools for the assessment of nausea. However, they were applied in only 20% studies included in this review for the assessment of nausea in pediatric CINV. A retrospective observational study by Flank et al concluded that more prospective, controlled trials using validated pediatric nausea assessment tools are necessary to determine the extent of the contribution of olanzapine to CINV control and its safety profile in this population.

Second, certain patient factors have been shown to increase the incidence of CINV (female, younger than 50 years of age, history of low but chronic alcohol consumption, history of chemotherapy-induced emesis, history of motion sickness and history of enuresis in past pregnancy).^[1,2]^ Conversely, pediatric patient factors remain poorly defined ^[22–25]^ and complicate the prediction of pediatric CINV risk that might help inform current antiemetic protocols.

Third, adult patient CINV management studies for single-day chemotherapy protocols often use complete control of emesis without the use of rescue medication as the primary endpoints over acute, delayed and overall CINV periods.^[1,2,23–25]^ However, most pediatric CINV studies use multi-day chemotherapy protocols making the evaluation of delayed CINV difficult as it can occur on any day of chemotherapy.^[23–25]^ In addition, there is uncertainty about the relative benefits and harms of different dosing schedules of olanzapine in the treatment and prevention of pediatric CINV. Equally, studies identified and included in this review provides evidence on the efficacy and safety of oral forms of olanzapine only and cannot be extrapolated to provide evidence on any injectable form (intravenous, intramuscular or subcutaneous) of olanzapine.

The differences in reported results might, in part, be explained by the variability of disease distribution within the pediatric cancer population across different ages and therefore, so does the chemotherapy used and emetogenic potential. There are also variations in treatment pathways worldwide (e.g., is an 18 years old with ALL treated on a “pediatric” style protocol or an adult-style leukaemia protocol), as well as variations in how a “child” is defined in practice. In addition, the variability of pharmacokinetic and pharmacodynamic properties of olanzapine across ages within the pediatric population pose additional challenges in establishing appropriate formulation and dose of olanzapine when developing pediatric treatment protocols.

The findings of this review help demonstrate the need for high quality research focusing on the safety of olanzapine in the control of pediatric CINV.

A study by Davis et al suggests that growing research on the efficacy of olanzapine in the treatment and prevention of pediatric CINV has led to a significant shift in prescribing practices over the last 4 years within the field of pediatric oncology that is away from the previously predominant combination of ondansetron and aprepitant towards olanzapine-containing regimens.^[38]^ However, the efficacy of olanzapine in the treatment of pediatric CINV needs to be carefully balanced against its drug safety profile. The most common side effect reported in the included studies was drowsiness which appeared to be dose-dependent followed by hyperglycemia. Other reported side-effects included gastrointestinal distress, constipation, abnormal liver enzymes, fatigue, sleep disturbance and agitation. Olanzapine is used in palliative care for the treatment of emesis, delirium, anxiety, insomnia, anorexia and cachexia in adults.^[6–8]^ The efficacy and safety of olanzapine in the management of physical (low appetite, weight loss) and psychological (anxiety, insomnia) symptoms associated with oncological conditions in children has not been well studied.

It is important to note that the majority of included studies for the purpose of this review originate from low and middle income countries where healthcare expenditure and resources are often limited. Olanzapine is a relatively inexpensive drug, compared to standard antiemetics used in management of CINV.^[5]^ Studies by Samaddar et al and Teotia et al argue that the low cost, oral formulation and safety profile of olanzapine has added value in cost-constrained settings.^[34,35]^

The major limitation of this review was the paucity of high-quality evidence. Most identified and included studies failed to reach statistical power due to relatively small sample size, absent external validity and likely biases. Studies differed significantly in their study design, outcome measures and methodology to allow for adequate comparison between them. Only 30% of identified studies were RCTs and 10% of RCTs were blinded. One half of identified studies where observational and 1 third had retrospective design.

## 5. Conclusion

The findings of this review indicate that the use of olanzapine in the control of CINV has not been well studied in children. The use of olanzapine has been restricted to children in whom other antiemetics have exerted very poor control of CINV. Modest evidence presented in this review suggests that the addition of olanzapine to the antiemetic treatment regimen in pediatric CINV is a worthwhile and cost-effective consideration. Notwithstanding, further high-quality research is needed to fully assess the therapeutic potential and versatility of olanzapine in pediatric CINV as well as pediatric oncology more broadly.

## Acknowledgments

Thank you to Mike Groszmann (Child & Adolescent Psychiatry Consultant, University Collage Hospital), Dr Simon Lewis (Child & Adolescent Psychiatry Consultant, University Collage Hospital) and Dr Carmen Soto (Paediatric Oncology Consultant, University Collage Hospital) for their support and input during this process.

## Author contributions

**Conceptualization:** Anabella Karla Barušić.

**Data curation:** Anabella Karla Barušić.

**Formal analysis:** Anabella Karla Barušić.

**Investigation:** Anabella Karla Barušić.

**Methodology:** Anabella Karla Barušić.

**Project administration:** Anabella Karla Barušić.

**Resources:** Anabella Karla Barušić.

**Software:** Anabella Karla Barušić.

**Validation:** Anabella Karla Barušić.

**Visualization:** Anabella Karla Barušić.

**Writing – original draft:** Anabella Karla Barušić.

**Writing – review & editing:** Anabella Karla Barušić.
